# Intimate partner violence, adverse childhood experiences and prenatal substance use in South Africa

**DOI:** 10.4102/sajpsychiatry.v28i0.1937

**Published:** 2022-12-09

**Authors:** Mathabo L. Ndumo, Busisiwe S. Bhengu, Sibongile Mashaphu, Saeeda Paruk, Andrew Tomita

**Affiliations:** 1 Department of Public Health Medicine, School of Nursing and Public Health, University of KwaZulu-Natal, Durban, South Africa; 2 Discipline of Psychiatry, School of Clinical Medicine, University of KwaZulu-Natal, Durban, South Africa; 3 Centre for Rural Health, School of Nursing and Public Health, University of KwaZulu-Natal, Durban, South Africa; 4 KwaZulu-Natal Research Innovation and Sequencing Platform (KRISP), College of Health Sciences, University of KwaZulu-Natal, Durban, South Africa

**Keywords:** adverse childhood experiences, HIV, substance abuse, pregnancy, IPV

## Abstract

**Background:**

Intimate partner violence (IPV) is one of the most pressing public health conditions among women worldwide, particularly in sub-Saharan Africa. Intimate partner violence in South Africa, along with human immunodeficiency virus (HIV), is an epidemic that is closely linked to trauma and substance use in women.

**Aim:**

This study aimed to identify factors associated with IPV among pregnant women, with a specific focus on adverse childhood experiences (ACE), prenatal substance use, and HIV status.

**Setting:**

A large public general hospital in the KwaZulu-Natal province.

**Methods:**

The sampled study participants included 223 adult postpartum women (18 – 45 years) based on convenience sampling who recently gave birth. Four separate logistic regression models were fitted to examine the role of ACE, perinatal substance abuse and HIV against threat (model 1), physical violence (model 2), sexual violence (model 3) and any IPV (model 4) outcomes (threat and/or physical and/or sexual violence).

**Results:**

The prevalence of threat, physical violence, sexual violence and any IPV were 19.7%, 16.6%, 1.8% and 20.2%, respectively. The total ACE scores ranged from 0 to 11 (of 13 possible events) with a mean of 3.28 (standard deviation [s.d.] = 2.76), where 14.4% reported using substances during pregnancy (*n* = 32) as well as 47.1% (*n* = 105) of participants living with HIV. The authors found that the presence of family support was protective against IPV physical.

**Conclusion:**

It is essential that obstetric services screen and address potential risk factors along the life course pathways from early adversity to adult maternal health that drive IPV, particularly in young women who may lack family support during pregnancy.

**Contribution:**

This research gives insight into the dynamics between IPV, HV, ACE and perinatal substance use facing young women in South Africa

## Introduction

Despite the considerable advances in human rights legislation in many countries,^1^ the widespread occurrence of intimate partner violence (IPV) remains an area of global concern.^1,2^ Intimate partner violence, defined by the World Health Organization (WHO) as behaviour within an intimate relationship that causes physical, psychological or sexual damage to those in the relationship,^1^ is a serious challenge in South Africa (SA). According to the most recent meta-analysis or systemic review, approximately half (~44%) of young women in sub-Saharan Africa (SSA) experience IPV.^3^ In SA, IPV is so widespread that it is considered the second-highest burden of disease after human immunodeficiency virus (HIV),^4^ which has had devastating effects and impacted young women negatively.^5^ In one study from SA, young women aged 16 – 24 years reported physical (80%) and sexual (67%) IPV over the previous 12 months’ period.^6^ In young mothers who are victims, IPV impacts both mother and child or children.^1^ It is believed that IPV victims may become perpetrators themselves, carrying out harmful acts on others, including their children.^7^

It is well-established that issues of HIV and IPV in SA are gendered and closely linked to young women. According to an HIV incidence study,^3^ girls in adolescence and young women are two times more likely to be infected with HIV than their male counterparts.^3^ In 2019, the Centre for Strategic and International Studies reported that almost 4500 South Africans are infected with HIV, and of those, 33% were adolescent girls and young women aged 15 – 24 years.^6^ While some of the reasons cited for HIV incidence among the different genders include multiple relationships, the age gap between partners power disparities,^4^ and violence against women and their vulnerability are also implicated.^3,8^ When women are threatened in a relationship, this disempowers them from using condoms, thus exposing them to further risk.^8^ In SA, women who had abusive or controlling male partners were at 50% increased risk of acquiring HIV than women who had not experienced IPV.^8^ Another link between HIV and IPV is that where violence is perpetuated, it may be difficult for the women who are victims to adhere to antiretroviral therapy (ART) or even disclose their HIV status, thus exposing them to increased risk of HIV infection.^4,9^

Many of the challenges experienced by pregnant women and risky behaviours are deeply rooted in early childhood beginnings. Adverse childhood experiences (ACEs) are linked to a myriad of health problems later in life and that include health risk behaviours, that women engage in from a young age.^10,11^ Adverse childhood experiences such as physical, sexual and emotional abuse and neglect are universally common and are associated with a number of poor health outcomes.^12^ These negative outcomes include risky health behaviours and either aggression or victimisation of one’s partner whether male or female.^13^ Adverse childhood experiences are, therefore, associated with the perpetration of IPV in adulthood.^10,14^ In light of gaps in the evidence from a HIV hyperendemic South African setting that considers early or distal to proximate factors in pregnant women, this study aimed to identify factors associated with IPV among this clinical population group, with a specific focus on ACEs, substance abuse during pregnancy, and HIV status.

### Aims and objectives

The study aimed to identify factors associated with IPV among pregnant women, with a specific focus on ACE, substance use, and HIV status. The study had the following objectives:

- To establish the association between IPV and ACEs.
- To calculate the association between IPV and prenatal substance use.
- To establish the association between IPV and HIV status.

## Methodology

### Study design and setting

The investigation involved a secondary data analysis of a cross-sectional study involving adult postpartum women who recently gave birth while attending one of the large public general hospitals in KwaZulu-Natal (KZN) province, South Africa.

### Study population and sampling strategy

The sampled study participants included 223 adult postpartum women (18 years – 45 years) based on convenience sampling.

### Data collection

The data collection took place between January and December 2018. The eligibility criteria were female participants who: (1) could read and/or write English or IsiZulu, (2) had given birth in the past week receiving in- or out-patient services at the hospital and (3) were aged between 18 and 45 years. All consenting study participants were interviewed by a senior psychiatry registrar fluent in English and IsiZulu. They were interviewed within one week of delivery using structured questionnaires.

### Measurement

The main outcome of the study was IPV experienced during a lifetime. There are four types of IPV outcomes^1,15,16,17^: (1) threat (e.g. threw, twisted your arm or hair, pushed, shoved or grabbed); (2) physical violence (e.g., were kicked, punched or hit, beat up, burned or scalded, choked); (3) sexual violence (e.g., were raped, or someone forced their private parts or object in the vagina or anal area) and (4) any IPV which refers to either threat and/or physical and/or sexual violence.

The main explanatory variable was ACEs, which were measured with the WHO’s Adverse Childhood Experiences International Questionnaire (ACE-IQ). It was previously used in other South African studies also.^3,15,16^ Adverse Childhood Experiences International Questionnaire consists of 13 categories of experiences during the first 18 years of life.

A clinical research form designed for this research was used to obtain self-reported status on any perinatal substance use. The HIV status was based on antibody testing, with two consecutive parallel tests using Architect^®^ (Abbott Diagnostics, Wiesbaden, Germany) and Cobas E601^®^ (Roche, Basel, Switzerland). Lastly, a sociodemographic questionnaire collected information on the participant’s age, occupation, education, social support, and income. Family support was measured by the presence of relationship with family members.

### Data analysis

Two analyses were undertaken for this investigation, the first summarising the participants’ sociodemographic and clinical profiles using descriptive statistics. The second examined the role of ACE, perinatal substance abuse and HIV against threat (model 1), physical violence (model 2), sexual violence (model 3) and any IPV (model 4) outcomes by fitting four separate logistic regression models. The data were analysed using Stata 17, and a *p*-value < 0.05 was considered statistically significant.

### Ethical considerations

Written consent was obtained from all participants, and the study was approved by the Biomedical Research Ethics Committee (Dual Approval #: BE479/17 and BREC/00003292/2021) at the University of KwaZulu-Natal.

## Results

### Sociodemographic profile

A total of 223 female study participants were involved in this investigation. Their mean age was 27.7 years (standard deviation [s.d.] = 5.52), most (*n* = 206; 92.4%) were black women, and 54 of the participants (24.2%) lived on an income of R1000.00 ($70.00) per month (Table 1).

**TABLE 1:** Sociodemographic and clinical characteristics of study participants (*N* = 223).

Sociodemographic and clinical characteristics	Variables	Overall			
		*n*	%	Mean	s.d.
Age	-	-	-	27.6	5.5
Occupation	Employed	82	36.8	-	-
	Unemployed	141	63.2	-	-
Income categories	< R1000.00	54	24.2	-	-
	R1001.00–R2500.00	61	27.4	-	-
	R2501.00–R5000.00	55	24.7	-	-
	R5001.00 and above	53	23.8	-	-
Education	Less than grade 12	66	29.6	-	-
	Grade 12	99	44.4	-	-
	Grade 12 and above	58	26.0	-	-
ACE	-	-	-	3.3	2.7
Pregnancy substance	No	191	85.7	-	-
	Yes	32	14.4	-	-
HIV	Negative	118	52.9	-	-
	Positive	105	47.1	-	-
Family support	No	62	27.8	-	-
	Yes	161	72.2	-	-
IPV threat	No	179	80.3	-	-
	Yes	44	19.7	-	-
IPV physical	No	186	83.4	-	-
	Yes	37	16.6	-	-
IPV sexual	No	219	98.2	-	-
	Yes	4	1.8	-	-
IPV any violence	No	178	79.8	-	-
	Yes	45	20.2	-	-

s.d., standard deviation; ACE, adverse childhood experiences; IPV, intimate partner violence HIV, human immunodeficiency virus.

### Clinical profile

The observed number of adverse events experienced in childhood ranged from 1–11 (of a possible 13 events), with a mean of 3.28 (s.d. = 2.76). Out of the 223 participants, 32 (14.4%) reported substance use during pregnancy, and 105 (47.1%) living with HIV. The prevalence of threat, physical violence, sexual violence, and any IPV, were 19.7% (*n* = 44), 16.6% (*n* = 37), 1.8% (*n* = 4) and 20.2% (*n* = 45), respectively (Table 1).

### Regression results

Table 2 summarises the logistic regression results against the IPV outcomes in: (1) threat, (2) physical violence, (3) sexual violence and (4) any IPV based on four separate fitted models. Except for sexual violence outcomes (model 3), ACE, perinatal substance abuse and HIV were all significant risk factors against threat (model 1), physical violence (model 2) and any IPV outcomes (model 4). The authors found that the presence of family support was protective against IPV physical. In model 1, ACE (adjusted odds ratio [aOR] = 1.53, 95% confidence interval [CI]: 1.28–1.83), perinatal substance (aOR = 4.55, 95% CI: 1.55–13.29) and HIV status (aOR = 4.19, 95% CI: 1.55–11.37) were significant risk factors for IPV threat. In model 2, ACE (aOR = 1.42, 95% CI: 1.18–1.70), perinatal substance (aOR = 5.67, 95% CI: 1.93–17.70) and HIV status (aOR = 2.90, 95% CI: 1.067.94) were significant risk factors for IPV threat. The authors found that the presence of family support was protective against IPV physical (aOR = 0.34, 95% CI: 0.13–0.90) in model 2. In model 4, ACE (aOR = 1.52, 95% CI 1.27–1.81), perinatal substance (aOR = 5.35, 95% CI: 1.84–15.50) and HIV status (aOR = 4.55, 95% CI: 1.69–12.30) were significant risk factors for IPV threat.

**TABLE 2:** Regression on the role on the roles of adverse childhood experiences, HIV and substance use during pregnancy on intimate partner violence (threat, physical, sexual, any) outcomes.

Variable	Category	IPV threat (Model 1)				IPV physical (Model 2)				IPV sexual (Model 3)				Any IPV violence (Model 4)			
		aOR	s.e.	95% CI		aOR	s.e.	95% CI		aOR	s.e.	95% CI		aOR	s.e.	95% CI	
Age	-	1.05	0.32	0.96	1.15	1.06	0.24	0.96	1.16	1.01	0.94	0.8	1.27	1.04	0.05	0.95	1.14
Occupation (Employed)	Unemployed	0.76	0.6	0.27	2.13	0.61	0.35	0.21	1.74	0.56	0.67	0.04	8.1	0.69	0.37	0.25	1.95
Income (R1000.00 or less)	R1001.00–R2500.00	0.86	0.79	0.27	2.74	0.56	0.33	0.17	1.82	0.82	0.9	0.04	19.04	0.85	0.50	0.26	2.72
	R2501.00–R5000.00	1.23	0.75	0.34	4.46	0.86	0.82	0.23	3.16	0.5	0.71	0.01	17.9	1.20	0.79	0.33	4.38
	R5000.00–R9999.00	0.09	0.02	0.01	0.67	0.10	0.03	0.01	0.80	0.21	0.45	0.01	12.93	0.13	0.12	0.02	0.86
Educational attainment (Lower than Grade 12)	Grade 12	1.26	0.67	0.44	3.63	0.98	0.97	0.34	2.86	0.36	0.51	0.02	7.59	1.21	0.65	0.42	3.47
	Grade 12 and below	0.75	0.69	0.17	3.22	0.62	0.53	0.14	2.75	3.32	0.45	0.15	74.9	0.85	0.63	0.20	3.62
ACE	-	1.53	0.01	1.28	1.83	1.42	0.01	1.18	1.70	1.38	0.15	0.89	2.16	1.52	0.14	1.27	1.81
HIV status (Negative)	Positive	4.19	0.01	1.55	11.37	2.90	0.04	1.06	7.94	0.59	0.66	0.05	6.55	4.55	2.30	1.69	12.28
Pregnancy substance (No)	Yes	4.55	0.01	1.55	13.29	5.67	0.01	1.93	16.66	5.06	0.19	0.44	58.01	5.35	2.91	1.84	15.52
Family support (No)	Yes	0.51	0.18	0.20	1.35	0.34	0.03	0.13	0.90	1.05	0.97	0.09	12.71	0.53	0.26	0.20	1.37

Note: Reference category in brackets.

IPV, intimate partner violence; HIV, human immunodeficiency virus; ACE, adverse childhood experiences; aOR, adjusted odds ratio; s.e., standard error; CI, confidence interval.

## Discussion

The authors investigated the role of adverse childhood experiences (ACEs), perinatal substance abuse, and HIV against IPV in one of the largest HIV hyperendemic settings globally. With the exception of sexual violence outcomes, the authors found, on the one hand that ACEs, perinatal substance abuse and HIV were identified to be consistent risk factors of IPV threat, physical and any IPV. On the other hand, the authors found that the presence of family support was protective against IPV physical.^17^ The authors’ findings on the role of ACEs, perinatal substance abuse and HIV against IPV are consistent with other major studies.^2,18,19^

The lack of significant findings against sexual violence warrants further discussion. A previous study by Kidman and Violari^20^ showed that ACEs, substance use during pregnancy and HIV were significantly associated with sexual IPV. The authors argue that the difference is because of the low prevalence of IPV sexual violence outcome. When adjusted logistic regression was fitted with fewer covariates, ACE and substance abuse in pregnancy against sexual IPV outcome was significant. For this reason, the findings on the sexual violence should be interpreted with caution.

Despite important study findings, there are two limitations worth noting. Firstly, this study is based on cross-sectional design, which limits causal inference and warrants further studies based on longitudinal methods that participants can be assessed early for any ACEs and tracked throughout their lifetime for any development of risky behaviours that could perpetuate HIV and IPV prevalence. Secondly, there could have been under-reporting of substance use during pregnancy. Unlike HIV status based on biomarkers, the study relied on self-report of substance use during pregnancy that is vulnerable to social desirability bias.

Notwithstanding these limitations, the strength of the study is the uniqueness of the sample. A group of women were approached within one week postpartum to have their voices heard. It is believed that ACEs are the root cause of risky behaviour that sustain the HIV epidemic,^21^ and the authors’ investigation attempts to make an important contribution to the body of knowledge about rarely examined essential role of the ACEs that fuel IPV epidemic in an HIV hyperendemic South African setting. We recommend that pregnant women seeking maternal care services in a hyperendemic HIV setting be screened for IPV and other psychosocial issues. They should receive holistic clinical services that address potential risk factors along the life course pathways from early adversity to adult maternal health that address risks factors. A special consideration should also be given to young women who may have insufficient family support during pregnancy.

## Conclusion

The study found that ACEs, perinatal substance use and HIV were associated with IPV. Considering that HIV is an endemic in KZN, this poses a threat to individuals’ health as well as the broader public health. The authors also found that the younger women are more prone to drug abuse and IPV. Findings show that family support was a protective strategy against physical IPV. Because of neglect and less utilisation of psychological services, family support was not found to be protective for other forms of IPV as it was with physical IPV.^22^ Therefore, further studies are needed.

Although there was no significant association between sexual IPV and ACEs, substance abuse and HIV, the authors conclude that it was because of the low prevalence of sexual IPV in the study, as this finding varied with other studies of a similar nature. The dynamics between IPV, HIV, ACEs and perinatal substance are of a complex nature. This requires more research into this field and increased awareness among the healthcare professionals and the public at large. The results of this research can be used to improve women’s quality of life by enhancing parenting strategies to avert ACEs. This will in turn, lessen the risk of being victims to IPV as girl children grow into womanhood.
